# Current Modulation of Plasmonic Nanolasers by Breaking Reciprocity on Hybrid Graphene–Insulator–Metal Platforms

**DOI:** 10.1002/advs.202001823

**Published:** 2020-11-17

**Authors:** Heng Li, Zhen‐Ting Huang, Kuo‐Bin Hong, Chu‐Yuan Hsu, Jia‐Wei Chen, Chang‐Wei Cheng, Kuo‐Ping Chen, Tzy‐Rong Lin, Shang‐Jr Gwo, Tien‐Chang Lu

**Affiliations:** ^1^ Department of Photonics College of Electrical and Computer Engineering National Chiao Tung University Hsinchu 30010 Taiwan; ^2^ Department of Physics National Tsing‐Hua University Hsinchu 30013 Taiwan; ^3^ Institute of Imaging and Biomedical Photonics National Chiao Tung University Tainan 71150 Taiwan; ^4^ Department of Mechanical and Mechatronic Engineering National Taiwan Ocean University Keelung 20224 Taiwan; ^5^ Research Center for Applied Sciences Academia Sinica Taipei 11529 Taiwan

**Keywords:** graphene, nanolasers, nonreciprocity, surface plasmon polaritons

## Abstract

A hybrid graphene–insulator–metal (GIM) platform is proposed with a supported surface plasmon polariton (SPP) wave that can be manipulated by breaking Lorentz reciprocity. The ZnO SPP nanowire lasers on the GIM platforms are demonstrated up to room temperature to be actively modulated by applying external current to graphene, which transforms the cavity mode from the standing to propagation wave pattern. With applying 100 mA external current, the laser threshold increases by ≈100% and a 1.2 nm Doppler shift is observed due to the nonreciprocal propagation characteristic. The nanolaser performance also depends on the orientation of the nanowire with respect to the current flow direction. The GIM platform can be a promising platform for integrated plasmonic system functioning laser generation, modulation, and detection.

## Graphical abstract

Plasmonic nanolasers are incorporated into the hybrid graphene–insulator–metal platform that can break the Lorentz reciprocity by applying external current to graphene in order to actively modulate the laser threshold. Room temperature laser modulation demonstrates that the laser threshold increases by ≈100% with applying 140 mA external current.

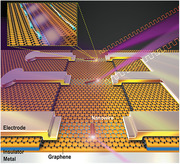

Breaking Lorentz reciprocity in optical components is indispensable for fully optical circuitry. The rapid development of integrated circuits is primarily attributable to the invention of the diode by the German physicist Ferdinand Braun in 1874, which provides the function of nonreciprocal current propagation.^[^ ^1^, ^2^, ^3^, ^4^ ^]^ In modern optics, several components have been proposed for use as optical diodes, isolators, and circulators in optical circuits based on the principle of nonreciprocity.^[^ ^1^, ^5^, ^6^, ^7^ ^]^ Previously, optical nonreciprocity was usually realized by breaking the symmetry in the electromagnetic properties of optical materials that generate a strong magnetic field; approaches included the Faraday effect,^[^ ^8^, ^9^ ^]^ Kerr effects,^[^ ^10^ ^]^ optical nonlinearity,^[^ ^1^, ^6^, ^11^, ^12^, ^13^ ^]^ and the topological quantum Hall effect.^[^ ^14^, ^15^, ^16^, ^17^ ^]^ However, the large size required for the magneto‐optical components and the strong external magnetic fields make establishing compatibility with on‐chip integrated circuit technology difficult.

Motivated by the development of nanophotonics, some studies have extended the concept of nonreciprocal optics to the surface plasmon polariton (SPP) system, which is a system for strongly coupling photons and surface plasmons that can drastically reduce their size to the sub‐micrometer scale and even break the diffraction limit.^[^ ^8^, ^10^, ^18^, ^19^ ^]^ Furthermore, ultrastrong mode confinement in the SPP system enables a strong interaction between light and matter, which is beneficial for realizing the nonreciprocal effect in a small footprint^[^ ^20^ ^]^ and direct electrical control in plasmonic nanolasers.^[^ ^21^ ^]^ The magneto‐optical effect in the noble metal–dielectric plasmonic system was investigated in 2007, and plasmon‐enhanced giant Faraday and Kerr effects were obtained in subwavelength hole array^[^ ^8^ ^]^ and slit array^[^ ^10^, ^18^ ^]^ Au films on the magnetic bismuth‐substituted yttrium iron garnet (Bi:YIG) layer. Unfortunately, the required external magnetic field is too strong to control the properties of surface plasmons to a degree sufficient for practical application. Recently, several theoretical studies have indicated the possibility of creating an SPP wave that propagates unidirectionally in a certain frequency range through the application of a longitudinal direct electric current on the metal surface; these electrons propagating along the current flow still couple with photons as moving sources of radiation, resulting in a Doppler shift of the SPP wave frequency. By contrast, conventional SPP waves respond to local oscillating electrons on a metal surface.^[^ ^22^, ^23^ ^]^ These theoretical works have shed light on using current instead of magnetic fields to create nonreciprocal optical components at a small scale.

To further minimize the size of optical components for the purpose of breaking Lorentz reciprocity by applying the external current, 2D materials, such as graphene, with the thickness of a single atom and a massless electron provided by the dispersionless Dirac cone band structure can effectively meet nanophotonic requirements.^[^ ^24^, ^25^, ^26^ ^]^ The mobility in graphene can be as high as 1.0 × 10^6^ m s^−1^,^[^ ^27^, ^28^, ^29^, ^30^ ^]^ which is beneficial for sustaining external current to break the symmetry in electromagnetic properties.^[^ ^31^, ^32^, ^33^ ^]^ Although the intrinsic plasma frequency of graphene is in the range of the middle‐to‐far infrared bands, combining graphene with noble metals can yield an excellent platform for supporting SPP waves in the near‐infrared, visible, and even near‐ultraviolet (UV) bands when the electrons in graphene and the metal surface simultaneously contribute to supporting plasmonic oscillation.^[^ ^34^, ^35^, ^36^ ^]^ We propose the design of a graphene–insulator–metal (GIM) platform, as shown in **Figure** **1**a, and demonstrated that its SPP propagation properties are influenced by graphene.^[^ ^37^ ^]^ In the configuration of the GIM platform, when the current is applied to graphene, the plasmon phase velocity can approach the Fermi velocity (*v*_f_ = *c*/300, where *c* is the speed of light in a vacuum) of the graphene by tuning the insulator thickness in the GIM platform;^[^ ^38^ ^]^ therefore, it is possible to generate nonreciprocal SPP waves to realize novel nanophotonic devices.^[^ ^39^, ^40^ ^]^

**Figure 1:**
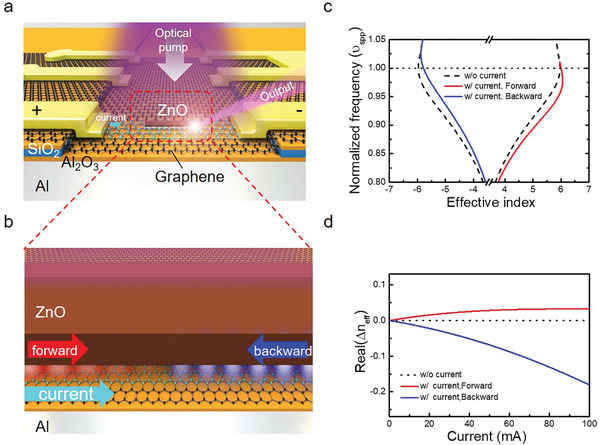
Propagation characteristics of surface plasmon polaritons (SPPs) beneath the ZnO nanowire on the graphene–insulator–metal (GIM) structure with external current applied to graphene. a) Schematic of a ZnO plasmonic nanolaser on the GIM structure when applying external current from the metal contacts to graphene and modulating the SPP wave propagating characteristics. b) Schematic of SPP wave propagation along the surface of the GIM structure beneath the ZnO nanowire. The propagation characteristics of forward‐ and backward‐propagating SPP waves along the nanowires were modulated by applying external current to graphene. c) The calculated dispersion curves of the mode in the structure of (a) for cases in which the external current was or was not applied. In the simulation model for a ZnO nanowire on the GIM structure, the side length of the ZnO nanowire was 35 nm, the thickness of the Al_2_O_3_ layer was 6 nm on the aluminium, and the thickness of the graphene layer was 0.5 nm. The positive and negative effective indices refer to forward and backward propagation direction, respectively. The dispersion curves of forward and backward propagation under current injection are not symmetric, and reciprocity is broken. d) The deviation of the effective index values of the mode in the structure of (a) was calculated as a function of applied current compared with the condition of no applied current. The dashed curves show the effective index without the application of external current, and the red and blue curves show the effective index of the SPP waves with forward and backward propagation directions with the application of an external current, respectively.

We first applied this current‐induced nonreciprocity in plasmonic nanolasers because the nanoscale coherent light sources are essential to building optical circuits and have recently undergone rapid development.^[^ ^41^, ^42^, ^43^, ^44^ ^]^ Whether the modulation of a laser to generate optical signals is direct or indirect is a key functionality in a photonic system. We recently demonstrated that characteristics of plasmonic lasers on the GIM platform can be greatly influenced by the graphene layer.^[^ ^37^ ^]^ Here, we detail the application of an external current on a graphene layer to create a nonreciprocal effect of the SPP wave in the plasmonic laser cavity beneath the ZnO nanowire and the unique formation of a Fabry–Perot cavity with travelling plasmonic waves; moreover, a Doppler shift of the plasmonic mode was unambiguously observed. Furthermore, modulation of the applied current on the graphene can modulate the intensity of the laser output, providing a viable approach for processing signals in plasmonic circuitry by using a GIM platform.

The proposed GIM plasmonic nanolaser is similar to the widespread semiconductor–insulator–metal nanolasers except an additional graphene layer is inserted between the ZnO nanowire and an Al_2_O_3_ separation layer, as shown in Figure 1a. To demonstrate the current‐modulated laser system, electrode pads were deposited on the GIM platform to apply external current to modulate the SPP characteristics. When an external current was applied, a nonreciprocal plasmonic phenomenon occurred; an illustration of the analogous mechanism for bidirectional SPP wave propagation is shown in Figure 1b. The propagation symmetry was broken because the interaction between the external current and SPP wave gave rise to a change in momentum (wave vector), which further yielded asymmetrical SPP mode properties such as effective index, propagation loss, and group velocity for forward‐ and backward‐propagating waves.

Figure 1c illustrates the results of the theoretical calculation of the dispersion curves with and without consideration of the current‐induced momentum change referred to as the Doppler frequency shift,^[^ ^22^ ^]^ which is the operational principle of the nonreciprocal phenomenon (see the Supporting Information). The appreciable difference between the forward‐ and backward‐propagating SPP waves is evident when the operating condition arises near the SPP resonant frequency *v_spp_* . This is attributable to the distinct effective index variation of the SPP wave near the resonance. The effective index deviation of the current‐modulated SPP waves was then calculated and compared with that of SPP waves at an identical excitation frequency but without the application of current, as shown in Figure 1d. With an increase in external current that accelerated the electron accompanying the energetic driving force, the calculated Doppler shift was dramatically increased. As indicated by the blue line plotted in Figure 1d, the deviation in the effective index of the backward‐propagating SPP wave was more significant than that of the forward‐propagating SPP wave, plotted by the red line, which resulted in a significant reduction in the effective index of the entire laser cavity mode. In such a pair of nonreciprocal SPP waves propagating in a Fabry‐Perot‐type cavity provided by a nanowire, the observation of an oscillating travelling wave in the cavity that fulfilled the round trip condition rather than a conventional standing wave (see the Supporting Information) was expected. In addition, the lasing wavelength would exhibit blueshift, and the threshold condition would be influenced with variation in the applied current. The strong nonreciprocal effect occurred when the effective index varied significantly. Although the operating frequency of the ZnO nanowire laser was considerably different from the plasma frequency of aluminium, the unique Lorentzian function of the ZnO exciton that featured a large oscillation strength and exciton binding energy^[^ ^45^, ^46^ ^]^ created a large effective index variation near the gain peak of ZnO and resulted in asymmetrical excitonic oscillations between forward‐ and backward‐propagating SPP waves.

**Figure** **2**a–e presents the scanning electron microscopy, transmission electron microscopy, and optical microscopy (OM) images of our samples at different magnifications. The sample was placed in a cryogenic chamber and optically pumped at 77 K. The electrodes on the graphene layer were connected to the electrode bars of the chamber, and the external current was provided by a continuous wave current supply through the wires on the exterior. The lasing behavior and the spectra of the ZnO nanowire laser applying external current are shown in Figure 2f,g. Before applying the external current, the lasing threshold was ≈77 µW; the sharp lasing peak could be observed with a pumping power above the threshold power. However, with the application of a 200 mA external current, the ZnO nanowire ceased to show any lasing signal, even as the pumping power reached 750 µW. By examining lasing peak intensity against pumping power, it is evident that the lasing threshold increased with current application. This provided sufficient evidence that nanowire lasing behavior can be modulated through application of the external current.

**Figure 2:**
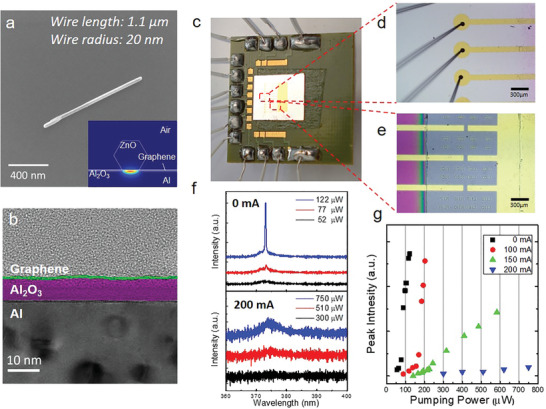
Sample structure and lasing properties of ZnO plasmonic nanolasers on graphene–insulator–metal (GIM) structures with external current injection. a) Scanning electron microscopy image of a ZnO nanowire on the GIM structure with a length and side length of ≈1.1 µm and 20 nm, respectively, for the hexagonal cross section of ZnO nanowires. The typical size range is ≈1–2 µm in length and 20–40 nm in side length. The inset shows the mode profile of the ZnO plasmonic nanolaser on the GIM structure without external current. b) Transmission electron microscopy cross‐section images of a GIM structure. The thickness of the Al_2_O_3_ layer is 6 nm, including a 4 nm native oxidation layer and a 2 nm Al_2_O_3_ layer deposited through atomic layer deposition. The surface roughness of the GIM platform is ≈1.13 nm, measured using an atomic force microscope. c–e) Optical microscopy image of the tested sample. The metal electrodes on the GIM structures were wire‐bonded to the PCB electrode pad and connected to an external current source. The gaps between yellow‐colored electrodes shown in (d) are trenches 70 µm wide in which ZnO nanowires were placed for measurement. f,g) Power‐dependent PL results measured from ZnO nanowires on GIM structures at 77 K, with and without current injection. The power‐dependent PL spectra are shown in (f), with and without current injection. g) The corresponding (a) light‐in versus light‐out (L–L) curves of the ZnO plasmonic nanolaser on the GIM structure under different conditions of current injection to the graphene. The thresholds of pumping power increased with the injected current.

To further confirm the characteristics of laser modulation by applying the external current and the physical mechanism, the external current alternated between forward and reverse flow to assess the lasing behavior; the repeatability test was also performed by switching the current on and off. As evident in **Figure** **3**a, regardless of whether forward or reverse flow was applied, the same effect was observed for ZnO nanowire lasers; the current increased in both the forward or reverse directions, causing the laser threshold to increase and inhibiting lasing behavior when the optical pumping power fixed at the two times threshold without current injected (2 × *P*_th,0mA_). However, laser intensity was recovered as the current decreased. Detailed analysis of peak intensity and position was performed through extraction and curve fitting, and the results are presented in Figure 3b. It is evident that the laser intensity was modulated by applying the current and that blueshift was evident with an increase in the external current; this is a signature characteristic of Doppler shift that is attributable to the nonreciprocal effect. The blueshifted wavelength was ≈0.3 nm when the applied current reached 150 mA. When the applied current was 200 mA, only the spontaneous emission signal could be observed in the spectrum; consequently, the peak position at 200 mA could not be determined.

**Figure 3:**
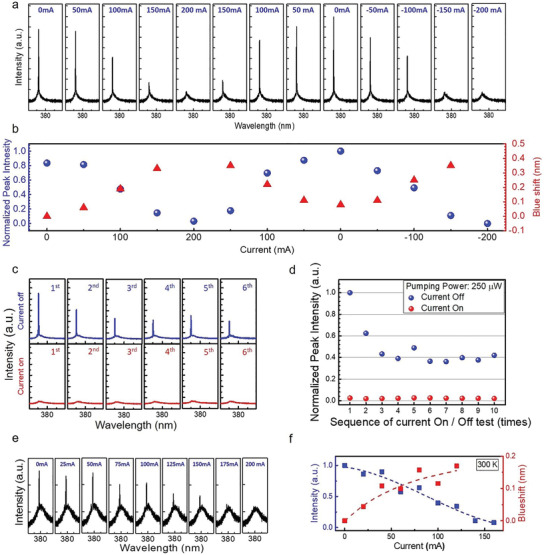
Characteristics of a ZnO plasmonic nanolaser on the graphene–insulator–metal (GIM) structure with external current injection. a) The lasing spectra of a ZnO plasmonic nanolaser on the GIM structure pumped at fixed power (2 × *P*_th,0mA_) were measured with varied current injection to graphene. b) The dominant peak intensity and detuned spectral positions with respect to the lasing peak under the condition of no current injection are indicated by blue sphere and red triangle, respectively, as a function of external current injection to graphene. As the injection current increased, whether positively or negatively injected, the peak intensity decreased; however, the lasing peak tended to exhibit blueshift. c,d) The repeatability test of current injection to the graphene was done by consecutively switching the current off and on between 0 and 200 mA while maintaining the same optical pumping level. The current switching period was ≈1 s, and the duty cycle was 50%. The lasing action was switched off completely every time the external current was injected. The individual lasing spectra are shown in (c), and the peak intensities are shown in (d), with their values normalized to the lasing intensity recorded initially when externally applied current was switched off. The lasing peak intensities under the current‐off condition decreased in the beginning and then remained constant afterward, indicating the repeatability of the system. e) The room‐temperature lasing spectra at the two times threshold (2 × *P*_th,0mA_) of a nanolaser on the GIM structure were measured with varied current injection from 0 to 200 mA. f) The dominant peak intensity and detuned spectral positions with respect to the lasing peak are indicated by blue and red squares, respectively, as a function of external current injection to graphene. A similar trend was observed at 300 K, that the peak intensity decreased and the lasing peak blueshifted.

Doppler shift is critical evidence of laser modulation through the nonreciprocal effect. However, other effects may influence laser behavior, including the thermal effect from the application of current and the field effects caused by a voltage difference between top and bottom metals. Both of these effects are isotropic; moreover, the heat effect is nonvolatile when the current or electric field is turned off. Figure 3c,d illustrates the repeatability test employed to investigate spectral variation; it entails switching the external current on and off between 200 and 0 mA. Upon application of a 200 mA current, no laser signals were observed. However, when the current was switched off, the laser intensity degraded to ≈40% of the maximum reached at the beginning and remained constant thereafter; this result is attributable to nonvolatile thermal accumulation during the current switching process.

The modulation mechanism of a ZnO plasmonic nanolaser on the GIM platform was also demonstrated at room temperature. In Figure 3e,f, the room‐temperature lasing spectra pumped at the two times threshold without current injected (2 × *P*_th,0mA_) of a nanolaser on the GIM platform were measured with varied current injection from 0 to 160 mA. A similar trend as the result measured at 77 K was observed at room temperature, that the dominant peak intensity decreased with external current increasing and the lasing peak vanished at about 140 mA external current applied. The Doppler shift, the critical evidence of lasing behavior modulated by the nonreciprocal effect, was also observed and the blueshifted wavelength was ≈0.15 nm when the applied current reached 120 mA. Briefly stated, this was clear demonstration that a plasmonic nanolaser can be modulated using external current and thus paves the way for the future development of plasmonic circuitry by using GIM platforms.

Because both thermal effects and field effects are independent of the orientation of nanowires, laser modulation with various nanowire orientations should be investigated with respect to current flow direction (see **Figure** **4**) to distinguish the nonreciprocal effect and other isotropic effects. The OM image shown in Figure 4b revealed the nanowire orientation of the light scattering from both ends of the nanowire above threshold condition. The lasing peak intensity and position of the ZnO nanowire on the GIM platform with different orientations were measured at fixed pumping power (2 × *P*_th,0mA_) and then fitted, as shown in Figure 4c,d. The pumping power was fixed at twice the threshold power of the ZnO nanowire on the GIM platform, without current injection for any wire. As shown in Figure 4c, an external current of ≈100 mA was applied to turn off the plasmonic nanolaser parallel to the current direction; however, a current exceeding 150 mA was required to turn off the lasing signals for the nanowires oriented at larger angles with respect to the current direction. The lasing peaks for plasmonic nanolasers oriented at different angles also exhibited obviously different blueshifted values. The maximum blueshift of 0.5 nm was obtained when the nanowire was parallel to the current. Because the nonreciprocal effect was strongest when the current was running parallel, the lowest current required to turn off these plasmonic nanolasers could be expected under this condition. Nevertheless, for the vertical nanowires, the lasing peak intensity of these plasmonic nanolasers was also degraded as the external current increased to a high level; this can be attributed to the heating effect. Notably, the lasing peak position of these vertical nanowires exhibited a minor blueshift compared with the obvious redshifted spontaneous emission peak of the ZnO nanowire as the current increased, as shown in the Supporting Information. This shift occurred because the threshold and the threshold carrier density increased with the heating effect induced by the external current and the effective refractive index decreased; thus, the lasing peak position exhibited a minor blueshift.^[^ ^47^ ^]^

**Figure 4:**
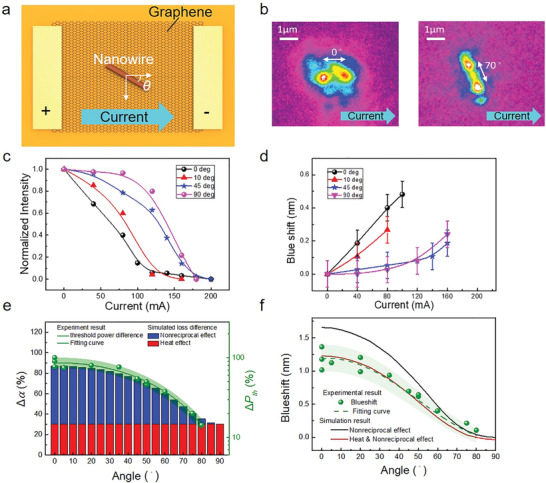
Nanowire angle‐dependent lasing properties of ZnO plasmonic nanolasers on the graphene–insulator–metal (GIM) structure with external current injection. a) Schematic of a ZnO plasmonic nanowire laser on the GIM structure rotated at an angle *θ* with respect to the current direction. b) The near‐field microscope images obtained above the threshold condition show the light scattered from two ends of the nanowires at different angles with respect to the current injection. c,d) The nanowire angle‐dependent lasing properties of ZnO plasmonic nanolasers on the GIM structure were measured when various external currents were injected at a fixed pumping power that was double the threshold value under the condition of no current injection (2 × *P*_th,0mA_). The peak intensities and detuned spectral positions with respect to the lasing peak under the condition of no current injection are shown in (c) and (d), respectively, where the ZnO nanowires are placed at *θ* = 0°, 10°, 45°, and 90° with respect to the current injection. The injection currents required to halt the lasing action and blueshift of the lasing peaks are obviously dependent on the angles of nanowires. When the nanowire is perpendicular to the current direction, the shut‐off current increases and a less blueshifted peak is obtained. e) The simulation model was constructed to analyze the physical phenomenon underlying the lasing behaviors and to compare the experimental laser threshold increasement between 0 and 100 mA external current injection as a function of angles of nanowires. In different nanowire angles, the simulated internal loss (Δ*α*) fits well with the change in threshold pumping power (Δ*P*_th_) and shows the logarithmic relationship. The green area depicted ±1*σ* error bars from the curve fitting of experimental data. The nonreciprocal effect and heat effect are considered. As the angle increases, the nonreciprocal effect gradually weakens but the heat effect remains. f) Simulated detuned spectral positions with respect to the lasing peak shift at the threshold under 0 and 100 mA injection as a function of the angles of nanowires. The green area depicted ±1*σ* error bars from the curve fitting of experimental data. The calculation results of blueshift of the lasing peaks are consistent with experimental results when considering the nonreciprocal and heat effects simultaneously. The nonreciprocal effect tends to blueshift the lasing peak, especially when external current is running parallel to the nanowire. However, the heat effect tends to offset the blueshift induced by the nonreciprocal effect.

Finally, the current‐modulated laser characteristics determined experimentally and through simulation, with various nanowire orientation angles *θ*_,_ are shown in Figure 4e,f. In the experimental results figure, the threshold increase and lasing peak blueshift were fitted and calculated from photoluminescence (PL) spectra with and without the application of external current. Each spectrum was measured at a pumping power that was 1.1 times higher than the threshold pumping power, and the applied external current was 100 mA. In the simulation model, the Doppler shift equation was adopted to represent the nonreciprocal effect to calculate the asymmetric dispersion of the SPP mode. Several other effects, including the redshift of gain peak and dispersion and the reduction of gain peak and line width broadening with the applied current, were considered in the gain medium, representing the heating effect. The wavelength of the SPP mode and variation of internal loss Δ*α* were determined by considering all effects in the modified resonant condition. A detailed discussion concerning the simulation model is provided in the Simulations of laser characteristics of the Experimental Section, and the Supporting Information.

Figure 4e depicts the change in the simulated internal loss (Δ*α*) and the measured increase in the threshold pumping power (Δ*P*_th_) with different nanowire orientation angles. When the nanowire was parallel to the applied current (*θ* = 0°), the nonreciprocal effect dominated the increase in internal loss and was approximately twice as strong as the heat effect, which indicated that the nonreciprocal effect becomes a significant loss mechanism in a current‐modulated laser system; the experimental data demonstrated that the threshold pumping power was doubled compared with the results without the application of current. The change in nanowire orientation directly affected the electron drift velocity and induced a cosine variation, Δ*α*. The nonreciprocal effect became weaker than the heat effect when *θ* was larger than 55° and completely vanished at 90°. In addition to the influence of internal loss, because the resonant condition with asymmetric mode dispersion was affected (Figure S1, Movies S1 and S2, Supporting Information), the broken phase of the standing wave resulted in the travelling wave resonance becoming another loss mechanism in the current‐modulated laser cavity. As the nanowire orientation angle *θ* increased, the change in the threshold pumping power (Δ*P*_th_) exhibited a consistent trend with the simulation result. As the external current increased, the change in threshold pumping power (Δ*P*_th_) decreased from 87% when the nanowire was parallel to the applied current (*θ* = 0°) to 14% when the nanowire was vertical to the applied current (*θ* = 0°). The change in threshold pumping power (Δ*P*_th_) exhibited a logarithmic relationship with the simulated internal loss (Δ*α*), and the simulation result was consistent with the experiment result.

Figure 4f depicts the blueshift of lasing peaks of plasmonic nanolasers, defined as the wavelength difference between conditions in which the current is off or on at 100 mA for different nanowire orientation angles in the experiment and simulation results. The simulation separately yielded results that did and did not account for the heat effect in the calculation model. The blueshift observed in the simulation results was fairly consistent with that observed in the experiment when the heat effect and nonreciprocal effect were taken into account in the simulation model; the maximum blueshift was ≈1.2 nm at 0°. We discovered that the blueshift decreased with the orientation angle of the nanowire because of the reduced nonreciprocal effect. The clear reduction in the blueshift value between the red and black curves in Figure 4f at a small angle was due to the strong nonreciprocal effect and a large slope variation in the dispersion relationship of ZnO when operating near the gain peak of the ZnO Lorentzian function. However, the nonreciprocal effect became weaker at a larger angle and completely vanished at 90°; a smaller setback between the red and black curves is expected at a larger angle. This angle‐dependent characteristic was not observed in the plasmonic nanolaser on the insulator/metal template with applying the current in metal (see the Supporting Information). As shown in Figure 4, this angle‐dependent laser characteristic unambiguously demonstrated a nonreciprocal effect on the GIM platform and strong potential for use in the design of on‐chip integrated plasmonic circuits with versatile functionality, such as SPP isolators, diodes, and modulators.

ZnO SPP nanolasers on a GIM platform were realized, and the function of current modulation was demonstrated. The high‐mobility graphene layer above the metal provided high‐velocity electrons to compete with the plasmonic phase velocity when external current was applied. We thus discovered that Lorentz reciprocity of plasmonic waves breaks and turns the plasmonic laser cavity mode from standing to traveling wave pattern. The nonreciprocity phenomenon was confirmed through both experimentation and simulation. In experiments at 77 K, an approximately twofold threshold power increase was observed with the application of a 100 mA external current when the ZnO nanowire was parallel to the current, and a 1.2 nm blueshift of the lasing peak due to the nonreciprocal propagation properties and Doppler shift was evident. At room temperature, the nanolasers can also be turned off by breaking the reciprocity at 140 mA current injection and 0.15 nm Doppler shift was observed. The nonreciprocal propagation effect and heat effect were both considered in the simulation model, which exhibited an excellent fit with the experimental results. Despite the fact that the nanowires required an optical pump to inject the carriers into the active region, the graphene in this GIM platform could serve as an electrical contact for the nanowires because it has been proven to be widely applicable in the use of transparent conductive films.^[^ ^48^ ^]^ The GIM platform not only demonstrated the breaking of Lorentz reciprocity and the Doppler effect in a nanolaser but also provides an effective method for modulating a nanolaser. This study demonstrated that the GIM platform plays a critical role in developing various novel active plasmonic devices necessary for realizing plasmonic circuitry.

## Experimental Section

##### Sample Preparation

First, a single‐crystalline Al film was grown through molecular beam epitaxy on the sapphire substrate. Detailed growth parameters of a single‐crystalline Al film were reported elsewhere.^[^ ^49^ ^]^ Atop the single‐crystalline Al film is an Al_2_O_3_ spacer layer, composed of a 4‐nm‐thick native oxidation layer and a 2‐nm‐thick Al_2_O_3_ layer deposited through atomic layer deposition (ALD). Next, the photoresist was applied and defined by the mask to create a 1.5‐mm‐wide trench. Subsequently, 520‐nm‐thick SiO_2_ and 80‐nm‐thick Al_2_O_3_ layers were deposited successively through plasma‐enhanced chemical vapor deposition (CVD) and ALD, which can prevent leakage caused by defects in dielectric layers. Graphene (1 cm × 1 cm) grown through CVD was then transferred onto the sample and across the trench by applying the wet transfer method. After graphene transfer, Au/Ti electrodes were deposited on the graphene by using an E‐gun. Twelve pairs of electrodes were employed, the width of each electrode was 100 µm, and the trench between anode and cathode electrodes was 70 µm. The sample was then attached to a plastic circuit board (PCB), and Au wires were bonded to the electrodes between the sample and PCB. Finally, ZnO nanowires, exhibiting hexagonal structures with typical sizes ranging from ≈1–2 µm in length and 20–40 nm in side length, were distributed in the trench.

##### Optical and Electrical Measurement

For measurement, samples were placed in a cryogenic chamber with a pressure below 10^−6^ bar. During sampling, the temperature was tuned from 77 to 300 K. The electric wires on the PCB were connected to the bars in the low‐pressure chamber, which was connected to the electric wires on the exterior of the chamber. Those wires were then attached to a power supply (Keithley 238) to separately provide DC current to each trench on the sample. ZnO nanowires on GIM structures were excited using a 355 nm third harmonic generation Nd:YVO_4_ pulse laser with a repetition rate of 1 kHz and a pulse duration of less than 0.5 ns. The laser beam was focused on the sample through a 100× near‐UV infinity‐corrected objective lens with a numerical aperture of 0.5 (Mitutoyo, Japan) and a working distance of 11 mm. The diameter of the focused spot was ≈15 µm. Special attention was paid to ensure that only one nanowire was pumped within the spot.^[^ ^37^ ^]^ Light emitted from ZnO nanowires was collected using a UV optical fiber with a core diameter of 600 µm and a UV collimating lens. The light was detected using a nitrogen‐cooled charge‐coupled device attached to a 320‐mm‐long single monochromator (iHR320, Horiba).

##### Simulations of Laser Characteristics

All simulation results of the dispersion curves, SPP mode profiles, effective indices, internal losses, and blueshift conditions of ZnO nanowires on the GIM structure with Al templates were obtained using a finite element mode analysis solver. The spontaneous coupling factor (*β*) of ZnO nanolasers with different applied currents was obtained using simplified rate equations. Otherwise, according to thermal accumulation attributed to the applied current, several effects were also considered into the dielectric function of ZnO nanowire to fit with the experimental results, including a redshift of the gain peak, gain attenuation, and linewidth broadening of the exciton oscillator (see Equation (S3), Supporting Information). In the case of an applied current of 100 mA, *δλ*_gain,r_ = 0.11 nm was chosen to simulate the results of Figure 4e,f, which represents the local temperature increased to ≈85 K with respect to the temperature‐dependent PL measurement. Details regarding calculation of the variation in internal loss and the wavelength shift in the current‐modulated laser systems are discussed in the first section of the Supporting Information and are presented in Figure S2 (Supporting Information).

## Conflict of Interest

The authors declare no conflict of interest.

## Supporting information

Supporting Information

Supplemental Movie 1

Supplemental Movie 2
