# Supplementary material for: Current Modulation of Plasmonic Nanolasers by Breaking Reciprocity on Hybrid Graphene–Insulator–Metal Platforms
Source: Adv Sci (Weinh). 2020 Nov 17;7(24):2001823. doi: 10.1002/advs.202001823 (PMC7740083; doi:10.1002/advs.202001823)
Supplement: Supplementary file 1 — Supporting Information [file ADVS-7-2001823-s001.pdf]

© 2020 Wiley-VCH GmbH

ADVANCED SCIENCE
Open Access

Supporting Information

for *Adv. Sci.,* DOI: 10.1002/advs.202001823

Current modulation of plasmonic nanolasers by breaking reciprocity on hybrid graphene-insulator-metal platforms

*Heng Li, Zhen-Ting Huang, Kuo-Bin Hong, Chu-Yuan Hsu, Jia-Wei Chen, Chang-Wei Cheng, Kuo-Ping Chen, Tzy-Rong Lin, Shang-jr Gwo, and Tien-Chang Lu**

Supporting Information

Current modulation of plasmonic nanolasers by breaking reciprocity on hybrid graphene-insulator-metal platforms

*Heng Li, Zhen-Ting Huang, Kuo-Bin Hong, Chu-Yuan Hsu, Jia-Wei Chen, Chang-Wei Cheng, Kuo-Ping Chen, Tzy-Rong Lin, Shang-jr Gwo, and Tien-Chang Lu**

Simulation models for current-modulated laser characteristics

This section discusses the theoretical method and simulation model of the current-modulated laser system. All the simulations are performed using the mode analysis solver of the finite-element method. Before an external current was applied to perturb the laser system, surface plasmon polariton (SPP) waves existed primarily between the Al and Al_2O_3 interface, as shown in the inset of Fig. 2b in the main text; the SPP waves propagate backward and forward along the nanowire cavity to match the resonant condition. After applying an external current, the propagation symmetry is broken; thus, the effective index, internal loss, and group velocity differ between the forward- and backward-propagated SPP waves, leading to the current-modulated laser system having a different resonant condition from the regular laser system. Therefore, we develop theoretical methods to calculate the current-modulated laser characteristics for ZnO plasmonic nanolasers on the graphene–insulator–metal (GIM) structure, which are discussed as follows.

First, the Doppler shift equation is used to calculate the permittivity of the modified materials; [1] this also leads to asymmetric mode dispersion (shown in Fig. 1b of the main text), which directly influences the effective index and internal loss between the forward- and backward-propagating SPP waves. The Doppler shift equation is expressed as follows:

$$\delta\omega_{\mathrm{Doppler}} = k_{\mathrm{spp}} u_{\mathrm{e}} \tag{S1}$$

where k_{spp} and u_e represent the propagation constant of the SPP wave and the electron drift velocity, respectively. The Doppler frequency shift $\delta\omega_{\mathrm{Doppler}}$ induced by the application of current is adopted to modify the material permittivity, represented as $\varepsilon(\omega)\rightarrow\varepsilon(\omega\pm\delta\omega_{\mathrm{Doppler}})$, where positive or negative frequency shift represents the forward- or backward-propagating wave with respect to the current direction. Subsequently, we clarify the current-modulated dispersion relationship on the basis of the modified material permittivity, resulting in asymmetric SPP propagation properties, by solving the eigenmodes, as shown in Fig. 1c of the main text. Notably, as the operation frequency nears plasmonic resonance, the permittivity of the modified material increases the difference in dispersion between the forward- and backward-propagating SPP waves. This phenomenon is evident in Fig. 1c in the main text, where the operation frequency is near the SPP resonant frequency.

Because SPP waves are present in the hybrid GIM platform, only part of the SPP wave interacts with the graphene layer, where the current is applied to accelerate the electrons. We assumed that applying an external current of 100 mA, which is related to the turn-off condition of the plasmonic nanolaser in the experimental measurement, would increase the electron drift velocity to half the value of the graphene Fermi velocity ($v_f \sim 1/120$ c, where c is the speed of light in a vacuum). [2-4] By taking into account the linear approximation of the electron drift velocity and Equation S1, the permittivity of the modified material with different applied currents can be determined. Moreover, the real part of the effective index deviation as a function of the applied current can be further calculated at the SPP resonant frequency, as shown in Fig.

1d in the main text. We also added a Lorentzian oscillator function $L(\omega)$, shown with respect to the ZnO material, to describe the energy transfer from the exciton to SPP oscillation.

$$L(\omega) = \frac{-A}{(\omega_g{}^2 - \omega^2) + i\omega\Gamma} \tag{S2}$$

where ω_g and A are the resonant frequency and the oscillator strength of the ZnO exciton or gain peak, respectively; Γ is the damping constant determined by the linewidth of the measured PL spectrum $\Delta\lambda$; ω is the response optical frequency; and ω_g is determined by the ZnO gain peak λ_g. In this study, we adopted A = 0.1, $\Delta\lambda$ = 3 nm, and λ_g = 371 nm in the Lorentzian oscillator function to represent suitable exciton oscillation properties.

Second, because the current applied in the graphene layer leads to thermal accumulation in the plasmonic laser system, the temperature of the system increases accordingly. In particular, the thermal accumulation and temperature increase of the ZnO nanowire induce several effects, including a dielectric function red shift due to bandgap narrowing, gain attenuation, and linewidth broadening of the exciton oscillator that influences the SPP wave characteristics in the laser system. First, the red shift of the ZnO nanowire in the dielectric function as temperature increases is represented as follows:

$$\varepsilon_{\text{ZnO,heat}} = \varepsilon_{\text{ZnO}}(\lambda - \delta\lambda_{\text{gain,r}}) \tag{S3}$$

Equation S3 expresses how the permittivity function of ZnO is red-shifted with respect to its dispersion relationship due to the heat, where ε_{ZnO} and $\varepsilon_{\text{ZnO,heat}}$ are the permittivity functions before and after consideration of thermal accumulation; $\delta\lambda_{\text{gain,r}}$ is the value of the ZnO gain peak after detuning due to the increase in temperature, which can be determined through experimentation with the applied current (as shown in Fig. S4: we chose $\delta\lambda_{\text{gain,r}} = 0.11\ \text{nm}$ to simulate the results of Fig. 4e and 4f in the main text, with an applied current of 100 mA). Gain attenuation and linewidth broadening of the ZnO exciton oscillator produces a temperature effect, which influences the oscillator strength and damping constant of the ZnO Lorentzian oscillator function. When the current is applied, we use $A_{\text{heat}} = A \times \exp^{-1}[(T_2 - T_1)/T_0]$ and $\Delta\lambda_{\text{heat}} = \Delta\lambda + \Gamma_{\text{ac}}(T_2 - T_1) + \Gamma_{\text{ex}}[e^{\frac{-E_a}{k_B T_2}} - e^{\frac{-E_a}{k_B T_1}}]$ to represent the effects of gain attenuation and linewidth broadening, where A_{heat} and $\Delta\lambda_{\text{heat}}$ are the modified oscillator strength and the linewidth after considering thermal accumulation, respectively; T_0, T_1, and T_2 are the characteristic temperature, operating temperature, and temperature after the current is applied; k_B is Boltzmann's constant; Γ_{ac} is the coupling strength of exciton–acoustic-phonon scattering; and Γ_{ex} represents impurity. The terms Γ_{ac} and Γ_{ex} are set at 74 μeV and 256.4 meV, respectively, according to the appropriate exciton dynamics of the ZnO nanowire. [5] E_a is the activation energy in the thermal quenching process. To establish good fit of the simulated calculation with the experimental results in Fig. 4e and 4f of the main text, E_a can be set at approximately 17.16 meV, which is in a reasonable range compared with the activation energy of ZnO quantum dots and nanocrystals. [6] T_0 is estimated to be 146 K, T_1 is 77 K, and T_2 can be determined according to the detuned wavelength shift $\delta\lambda_{\text{gain,r}}$. In the case of an applied current of 100 mA, because $\delta\lambda_{\text{gain,r}} = 0.11\ \text{nm}$, T_2 is estimated to be approximately 85 K.

Finally, to calculate the oscillation condition of the laser cavity, the round-trip resonant condition is utilised, as expressed in Equation S4.

$$2k_0 n_{\text{eff}} L + 2\phi = 2\pi q = \frac{4\pi L}{\lambda_{\text{eff}}} \tag{S4}$$

where k_0, L, and q are the propagation constant in vacuum, cavity length, and mode number, respectively; and ϕ is the phase shift of the normal reflection at the cavity-to-air interface, which is primarily dependent on the effective index n_{eff} of the SPP wave. In the simulation model, an L value of 1.64 μm is selected because it is a typical nanowire length in our experiments. The operational wavelength is set at 370.85 nm, which was estimated by averaging the lasing wavelength observed from the experimental PL spectra. At the operational wavelength, n_{eff} can be obtained from the calculation of the mode dispersion relationship value, which is 1.685; the corresponding mode number is 15. Furthermore, the internal loss, which is derived from the imaginary part of the effective index, and ϕ are calculated to be approximately 0.4345×10^{5} cm^{-1} and 0.1374 rad. We then define an effective wavelength λ_{eff} to represent the resonant condition without its reflection phase shift. It is useful to investigate the variation in the lasing mode under different resonant conditions (with and without applying current) at a fixed λ_{eff} for same mode number. By using Equation S4, λ_{eff} is determined to be 219.5 nm at the operational wavelength. After the propagation symmetry is broken by the applied current, Equation S4 can be used to derive Equation S5.

$$\lambda_{eff} = \frac{\lambda}{\frac{n_{eff,+}+n_{eff,-}}{2}+\frac{\phi_{+}+\phi_{-}}{4\pi L}\lambda} \tag{S5}$$

where + and − respectively represent the forward and backward propagation direction of the SPP waves. When no external current is applied to the graphene layer, propagation symmetry is evident in the SPP wave and the following effective index and phase are equal: $n_{eff,+}=n_{eff,-}$ and $\phi_{+}=\phi_{-}$. After applying an external current to the graphene layer, the propagation symmetry is broken and $n_{eff,+}$, $n_{eff,-}$, ϕ_{+} , and ϕ_{-} can be determined from the asymmetric mode dispersion relation calculated using the Doppler shift equation. Subsequently, λ_{eff} , which is the function of the wavelength, can be also determined. Under the condition of fixed λ_{eff} , the modulated resonant wavelength can be estimated to demonstrate the wavelength shift phenomenon under different resonant conditions. In our simulation, we calculate the current-modulated resonant wavelength to be 369.62 nm by taking both the nonreciprocal effect and thermal accumulation into account at the fixed λ_{eff} and the corresponding $n_{eff,+}$, $n_{eff,-}$, ϕ_{+} , and ϕ_{-} at 1.754, 1.596, 0.2455 rad, and 0.2531 rad, respectively. Under such conditions, the wavelength is blue-shifted by approximately 1.23 nm when an external current is applied to the GIM laser cavity; the detailed variation of internal loss is shown in Fig. S2.

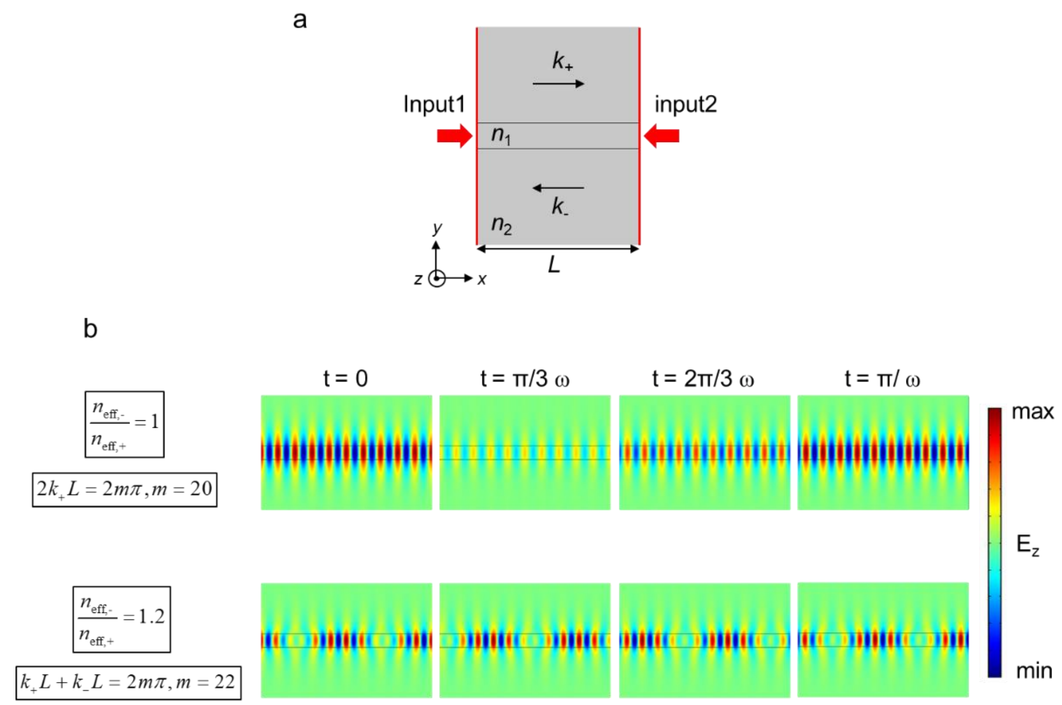

Fig. S1. Wave oscillation in a Fabry–Perot cavity under reciprocal and nonreciprocal conditions. (a) Schematic of the simulation model of a Fabry–Perot cavity, where L is the cavity length, n_1 is a waveguide with a core refractive index, and n_2 is a cladding refractive index. Two fundamental guided modes are incident in opposite directions from two boundaries of the Fabry–Perot cavity and investigate the interference patterns. (b) The real part of the electric field for the z component of counterpropagating modes with different effective index ratios oscillating with time when the simulation is neglecting the phase shift of the reflection.

To demonstrate the variation of the resonance in the Fabry–Perot cavity when the propagation symmetry is broken, we use a simple model entailing a waveguide with two counterpropagating guided modes to form an interference pattern of a finite length to mimic the resonance in the laser cavity. The schematic is shown in Fig. S1a. The effective indices of two guided modes for input beams 1 and 2 at two facets of the cavity are denoted as $n_{eff,+}$ and $n_{eff,-}$. To mimic the nonreciprocal propagation characteristics, the refractive index ratio n_2/n_1 of the core and cladding is modified to modulate the effective index ratio $n_{eff,+}/n_{eff,-}$ of the two incident guided modes. Because the length L is fixed, to satisfy the round-trip condition, we arbitrarily set the mode number m equal to 20 when the effective index ratio is equal to 1 to represent the conventional symmetric propagation condition. As shown in the upper row of S1b, a clear standing wave profile can be observed, demonstrating that the fields oscillate locally with time. By contrast, as shown in the lower row in S1b, when $n_{eff,+}/n_{eff,-}$ is set to 1.2 to mimic the asymmetric guided mode inputs, the standing wave patterns disappear and noticeably transform into travelling wave form and the mode number m changes to 22 to fulfil the round-trip condition. The phase of the reflective coefficient at the facet remains constant in this simple model. In the real plasmonic cavity of the ZnO nanowire SPP waves on the GIM platform, applying current parallel to the nanowire direction causes the nonreciprocal effect to also result in phase variation at the facet of the nanowire. The detailed findings of wave oscillation under reciprocal (Movie 1) and nonreciprocal (Movie 2) conditions are shown in the supplementary movie clips.

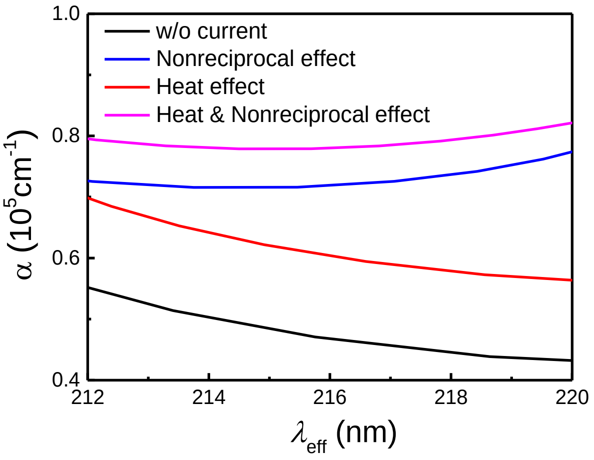

Fig. S2 Calculated internal loss with different effective wavelengths. The internal loss α is calculated by averaging the internal loss of the forward- and backward-propagating SPP waves in the ZnO nanowire cavity, and the effective wavelength is determined using Eq. S5, which considers the operating wavelength, effective index, and phase shift of reflection. By taking into account all of the conditions discussed in the part 1 of this supplementary information and assuming the nanowire length is 1.64 μm when the current is in parallel to the nanowire, the effective wavelength is calculated to be 219.5 nm. The corresponding internal loss is calculated to be $\alpha \approx 0.4345 \times 10^5 \text{cm}^{-1}$ in the case when no current is applied, $\alpha \approx 0.5669 \times 10^5 \text{cm}^{-1}$ when applying current but only considering the heat effect, and $\alpha \approx 0.8130 \times 10^5 \text{cm}^{-1}$ when applying current and considering both the nonreciprocal effect and heat effect.

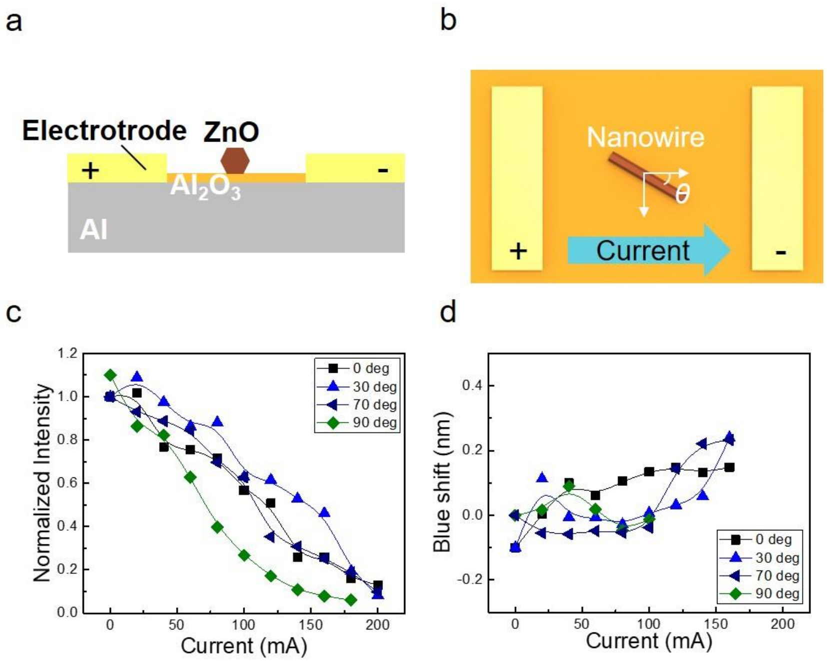

Fig. S3 Structure schematic and nanowire angle–dependent lasing properties of ZnO semiconductor-insulator-metal (SIM) plasmonic nanolasers. (a) Schematic of a ZnO SIM nanolaser with metal contacts for applying external current. **(b)** Schematic of a ZnO plasmonic nanowire laser with the SIM structure rotated at an angle θ with respect to the direction of current. **(c)** The current-dependent peak intensity and **(d)** blue shift of different nanowire orientations measured at a fixed pumping power that is twice the threshold pumping power when no current is applied. Evidently, the turn-off injection current is not dependent on the orientation of nanowires, and neither is the blue shift when current is applied. Plasmonic lasing ceases when current is applied primarily because of the heat effect, which is purely isotropic. The slight blue shift of the lasing peaks when higher current is applied is due to the Moss–Burstein effect insofar as the threshold and carrier density increase with the application of current. Here, we demonstrate that graphene plays an important role in providing rapidly moving electrons, resulting in a significant nonreciprocal effect. When the current only flows in the metal layer, the nonreciprocal effect is too weak to induce a change in threshold and the lasing wavelength. Therefore, the heat effect dominates the lasing operation in the ZnO plasmonic nanowire laser with an SIM structure.

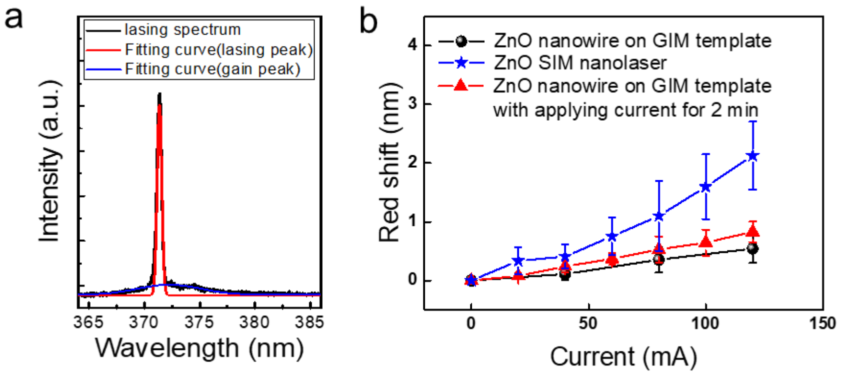

Fig. S4 Photoluminescence spectra of ZnO nanowires and spontaneous emission peak variation when external current is applied. (a) Photoluminescence spectra measured from a ZnO nanowire at 77 K. The lasing peak and spontaneous emission peaks are separately fitted. The spontaneous emission peak is labelled as the gain peak of the ZnO nanowire. **(b)** Spontaneous emission peaks of ZnO nanowires on the GIM platform and the ZnO SIM nanolaser vary with the applied current. All three cases exhibit the trend of red shift with increase in current. The red-shifted spontaneous emission peak results from the bandgap narrowing of the ZnO nanowires due to the heating effect caused by the current. The red shift of the ZnO SIM nanolaser (blue line) is clearly greater than that of the ZnO nanowires on the GIM platform, indicating that the insertion of a graphene layer can be effective for heat dissipation. [7] The red line and black line are the spontaneous emission peak shifts of the ZnO nanowires on the GIM platform measured after applying an external current for 2 and 0 minutes, respectively. A slightly higher red shift of the red line compared with the black line is evident, reflecting the effect of heat accumulation resulting from steadily applied external current.

Movie S1.
The wave oscillation in a Fabry-Perot cavity under reciprocal conditions (AVI).

Movie S2.
The wave oscillation in a Fabry-Perot cavity under nonreciprocal conditions (AVI)

References

[1] K. Y. Bliokh, F. J. Rodríguez-Fortuño, A. Y. Bekshaev, Y. S. Kivshar, F. Nori, *Opt. Lett.* **2018** *43*, 963-966.

[2] B. V. Duppen, A. Tomadin, A. N. Grigorenko, M. Polini, *2d Mater.* **2016** *3*, 015011.

[3] T. Wenger, G. Viola, J. Kinaret, M. Fogelström, P. Tassin, *Phys. Rev. B* **2018** *97*, 085419.

[4] C. Hwang, D. A. Siegel, S.-K. Mo, W. Regan, A. Ismach, Y. Zhang, A. Zettl, A. Lanzara, *Sci. Rep.* **2012** *2*, 590.

[5] Y. Zhang, D.-J. Chen, C.-T. Lee, *Appl. Phys. Lett.* **2007** *91*, 161911.

[6] V. A. Fonoberov, K. A. Alim, A. A. Balandin, F. Xiu, J. Liu, *Phys. Rev. B* **2006** *73*, 165317.

[7] M.-H. Shih, L.-J. Li, Y.-C. Yang, H.-Y. Chou, C.-T. Lin, C.-Y. Su, *ACS Nano* **2013** *7*, 10818-10824.
